# Long-Term Stability of TiS_2_–Alkylamine Hybrid Materials

**DOI:** 10.3390/ma15238297

**Published:** 2022-11-22

**Authors:** Federica Ursi, Simone Virga, Gonzalo Garcìa-Espejo, Norberto Masciocchi, Antonino Martorana, Francesco Giannici

**Affiliations:** 1 Dipartimento di Fisica e Chimica—Emilio Segrè, Università di Palermo, Viale delle Scienze, 90128 Palermo, Italy; 2 Dipartimento di Scienza e Alta Tecnologia and To.Sca.Lab., Università dell’Insubria, Via Valleggio 11, 22100 Como, Italy

**Keywords:** chalcogenides, thermoelectrics, X-ray diffraction, Raman, intercalation

## Abstract

Layered TiS_2_ intercalated with linear alkylamines has recently attracted significant interest as a model compound for flexible *n*-type thermoelectric applications, showing remarkably high power factors at room temperature. The thermal and, particularly, environmental stability of such materials is, however, a still an open challenge. In this paper, we show that amine-intercalated TiS_2_ prepared by a simple mechanochemical process is prone to chemical decomposition through sulfur exsolution, and that the presence of molecular oxygen is likely to mediate the decomposition reaction. Through computational analysis of the possible reaction pathways, we propose that Ti-N adducts are formed as a consequence of amine groups substituting for S vacancies on the internal surfaces of the S-Ti-S layers. These findings provide insights for possible future applications of similar hybrid compounds as devices operating in ambient conditions, and suggest isolating them from atmospheric oxygen.

## 1. Introduction

Thermoelectric (TE) materials are attracting increasing attention in the materials research community as they allow for the direct conversion of waste heat into electricity, thereby improving the overall efficiency of power generation [1,2,3,4]. Typically, thermoelectric devices have been so far based on inorganic compounds, but also hybrid (organic-inorganic) TE materials have recently attracted the attention of many, in both the academic and industrial fields [5,6,7,8,9]. Indeed, considering the exponential increase in energy consumption, the possibility to recover electrical energy directly from lower-temperature waste heat using hybrid materials may represent a crucial advantage over inorganic ones [10]. Moreover, TE devices fed by the heat produced during metabolic processes are considered today a promising energy source for wearable electronics [1], such as fitness trackers, smartwatches, and medical sensors. This latter class of devices requires the simultaneous fulfilment of (at least) two conditions: a good TE efficiency and a suitable fitting to the human body (as films, patches, or clothes), as allowed by flexible TE materials. In this line, we foresee that hybrid TE materials, combining the good electrical transport properties of semiconducting transition metal dichalcogenides (TMDC) with the typically low thermal conductivity of organic compounds, might be considered promising candidates, particularly if the solution is processable and cast as thin films [3,11].

Our interest in TE applications of TMDC focuses on those species that crystallize in layered structures. In these cases, the basic stoichiometry is MX_2_, where M is a transition metal, and X is a chalcogen: S, Se, or Te. These materials consist of tightly and covalently bonded X-M-X stacks or sheets running normal to one crystal axis (usually taken as the c-direction); at variance, the stacking sequence and geometry are stabilized by weak(er) van der Waals (vdW) interactions between the highly polarizable X atoms of adjacent layers. For what it matters here, one of the most interesting properties of TMDC is their ability to act as host lattices and, consequently, to interact with a wide variety of guest atoms or molecules to give rise to the formation of intercalation compounds [12,13,14]. Different synthetic methods for the preparation of such hybrids exist (liquid permeation, exfoliation, etc.), which have been beautifully reviewed by Guo et al. and Jung et al. [15,16]. Among layered TMDC, titanium disulfide (TiS_2_, the archetype of 2D transition metal dichalcogenides) is a promising candidate for *n*-type thermoelectrics owing to some remarkable properties: it is chemically stable, mechanically manageable [17], and environmentally benign and contains earth-abundant elements (Ti and S).

Intercalation compounds of TMDC, where neutral organic molecules (NOM) are inserted in a regular or less ordered fashion, have been the subject of numerous studies [13,18,19,20]. However, many relevant aspects, such as stoichiometry, chemical stability, thermal inertness, polymorphic occurrence, or the nature of MX_2_-NOM bonding, are still not completely clarified.

In this paper, we report the synthesis and both the physicochemical and the structural characterization of TMDC–alkylamine intercalation compounds obtained by a simple and reproducible, green and solventless mechanical grinding process, which fulfills the atom economy protocol [14,21].

Based on the experimental evidence of elemental sulfur formation from the TiS_2_–hexylamine hybrid upon aging in environmental conditions (an obvious drawback for ambient use and exposure of this material), we present an ab initio DFT study and evaluate the formation enthalpy of the several species that could explain possible reaction paths toward the (undesired) formation of the crystalline (orthorhombic) S_8_ species. In this way, the most probable decomposition mechanism is pinpointed, paving the way for proper countermeasures to be taken into account for practical applications.

## 2. Materials and Methods

### 2.1. Synthesis

**Figure 1:**
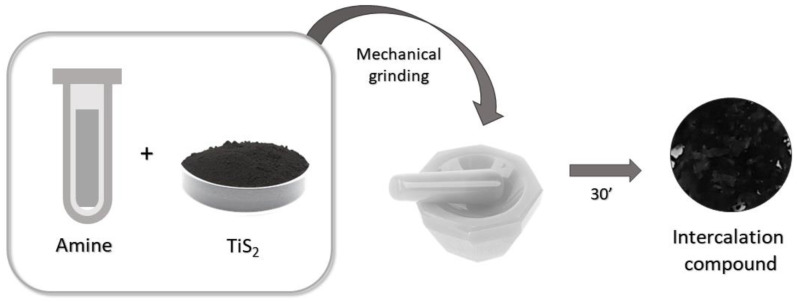
Scheme of the intercalation process through mechanochemical synthesis.

TiS_2_ powder (99.8% metals basis, Alfa Aesar, Haverhill, MA, USA) and liquid *n*-hexylamine (99%, Sigma-Aldrich, St. Louis, MO, USA, labeled as HA in the following), combined in a 1:4 molar ratio, were mixed in an agate mortar and manually ground with a pestle for 30 min (Figure 1). This synthesis was found to be highly reproducible. After grinding, the volume of the powders expanded significantly, indicating that the intercalation was effective, with an evident color change from black to shiny brown. The intercalated hybrid is labeled as TiS_2_/HA below. The pertinent chemical reaction then reads:

TiS_2(s)_ + x [n-C_6_H_13_NH_2(l)_] → TiS_2_(C_6_H_13_NH_2_)_x(s)_ with x ≈ 1 (1)

This material was found to be unstable if exposed for a prolonged time to environmental conditions. Indeed, we noticed that after 12 months storage without specific precautions, TiS_2_/HA had gone through macroscopic changes. For this reason, the effect of aging on this sample was further investigated. Two batches of freshly prepared TiS_2_/HA were left for 1 week in different environments: one in air and the other in inert atmosphere (N_2_), both in the dark and at room temperature.

### 2.2. Experimental Characterization

X-ray powder diffraction (XRPD) data were acquired in Bragg–Brentano geometry on a Rigaku Miniflex 600 (Tokyo, Japan) or on a Bruker D8 Advance diffractometer (Billerica, MA, USA), both working in vertical scan using Ni-filtered Cu K_α_ radiation. XRPD traces were analyzed with Topas [22], for peak hunting whole pattern profile analyses, in the structureless (Le Bail) or Rietveld modes (see Supplementary Materials for the full description of such analyses).

Thermogravimetric traces under nitrogen were acquired from room temperature to 700 °C using a TA Q500 thermogravimetric analyzer (New Castle, DE, USA) with a Pt sample holder. Micro-Raman spectra were recorded on a Horiba Raman Evolution spectrometer employing a confocal microscope with 50× long working distance and a laser with an excitation wavelength of 633 nm. Imaging by scanning electron microscopy (SEM) was performed in secondary electron mode using FEI Versa 3D (Lincoln, NE, USA) using a 10 kV acceleration voltage.

### 2.3. Computational Methods

Periodic ab initio DFT + U calculations were carried out with the PWscf package in the Quantum ESPRESSO 6.7 suite [23,24]. TiS_2_ hybrids were modeled starting from the *P-3m1* trigonal parent structure, and enlarging the *c* lattice parameter to accommodate amines. All structures were fully relaxed, and energies were calculated using a k-space sampling over a dense 8 × 8 × 4 grid. The pseudopotentials and kinetic energy and density cut-offs were taken from the standard solid-state pseudopotential efficiency library (SSSP) [25,26]. All calculations were performed using the generalized gradient approximation [27] and the PBEsol exchange-correlation functional [28]. The value of Hubbard U parameter for the Ti atom was set to 3 eV [29]. The van der Waals interlayer interactions were considered using Grimme’s D2 dispersion correction [30,31].

## 3. Results and Discussion

**Figure 2:**
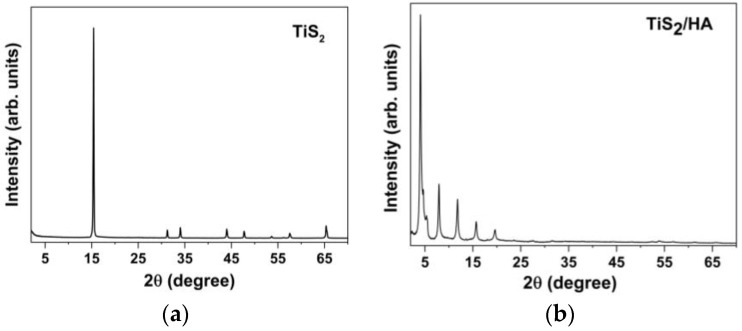
XRPD patterns of (**a**) pristine TiS_2_ and (**b**) TiS_2_/HA.

The macroscopic evidence of materials swelling during synthesis clearly indicated that hexylamine could be rapidly incorporated into TiS_2_ by a simple mechanochemical synthetic method. This unsophisticated method was indeed quantitative and could be repeated many times with the same outcome, providing robust information on its reproducibility. The structural changes were easily followed by acquiring X-ray powder diffraction (XRPD) data from the final product. In this sense, XRPD was first used in its qualitative (i.e., fingerprinting) mode, and when later used in a quantitative way, it allowed the determination of the axial *d*-spacings of the intercalated materials and for assessing either the presence of contaminant residues or the (unexpected) formation of elemental sulfur upon sample degradation. Specifically, in the pristine TiS_2_ solid, where Ti^4+^ ions are sandwiched between two sulfide layers, the 001 peak, corresponding to the stacking periodicity, falls near 15.7° (i.e., 5.69 Å). After 30 min grinding, when the macroscopic alterations exhibited by the powders (liquid amine absorbed, volume increment, and color change) indicated that the intercalation of hexylamine into TiS_2_ occurred, XRPD was used to monitor the changes in the interlayer distance between adjacent S-Ti-S sheets. In the TiS_2_/HA species, the 001 peak in the XRD pattern (the strongest one in the traces shown in Figure 2) shifted from 15.7° to 4.1°. This implies an enormously increased (4×) separation between layers (21.6 Å) compared with that found on the pristine TiS_2_ (5.69 Å), confirming HA intercalation.

It is worth noting that the stacking sequence in polymorphs and polytypes of intercalated TiS_2_ can be different, and is normally addressed by the occurrence of superstructure peaks, here not observed. However, since we detected peaks along only one reciprocal space rod *(00l)*, this technique may be fully blind to polymorphs occasionally sharing the same d-spacing along **c**. Thus, we cannot exclude that the TiS_2_ layers in the intercalated hybrids are slightly offset in the *xy* plane.

**Figure 3:**
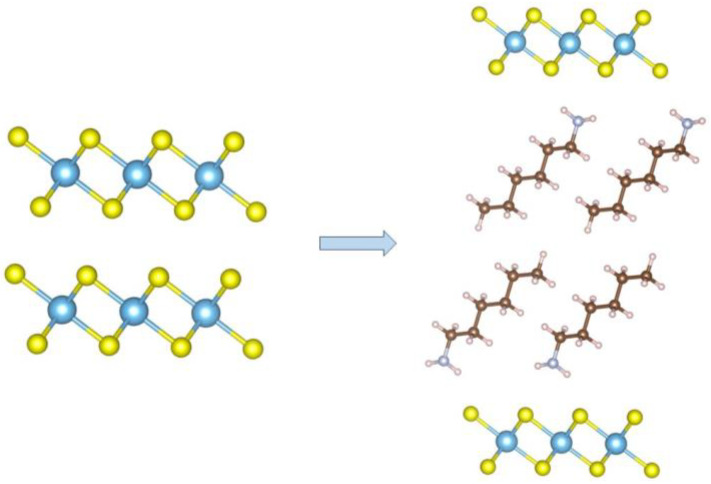
Left: crystal structure of pristine TiS_2_. Right: proposed crystal structure of TiS_2_/HA, containing one HA molecule per TiS_2_ formula unit.

Considering the c-axis expansion (ca. 15.9 Å), we can safely conclude that HA was embedded into the TiS_2_ lattice, forming a bilayer structure. Since the estimated length of a single HA molecule, in its common all-trans conformation, H-bonded to S and van der Waals radii-corrected, is ca. 11.5 Å, the limited increase of the c-axis value suggests that the intercalated molecules possess a measurable inclination in the van der Waals gap of TiS_2_, being 44° the estimated angle: sin^−1^(15.9/(2 × 11.5)) = 43.7° (Figure 3). Our DFT computational analysis provided geometry optimization of the HA location and orientation, eventually leading to an inclination of 40.0°, in very good agreement with the purely geometrical consideration set above. Density and geometrical considerations also indicate that 100% filling of the interlayer separation requires a stoichiometric TiS_2_/HA formulation (one amine per TiS_2_ unit formula), which, inter alia, would provide a cross-section area of ca. 20.0 Å^2^, like that found in the orthorhombic all-trans polyethylene crystal phase [32].

Thermogravimetric studies (TGA) were performed to estimate the quantity of hexylamine in the inorganic matrix, through calculation of the molar coverage fraction (as mol/mol TiS_2_), defined as:

(2) $$\frac{\Delta {\%}_{\mathrm{org}}\times \mathrm{MM}\left({\mathrm{TiS}}_{2}\right)}{\mathrm{MM}\left(\mathrm{HA}\right)\times \left(100-\Delta {\%}_{\mathrm{org}}\right)}$$

where Δ%_org_ is the weight loss at 250 °C due to the organic moieties, and MM are the molar masses.

The weight loss of pristine TiS_2_ was 3.36%, which was subtracted from Δ%_org_. To obtain a reproducible result, the sample was prepared and subjected to TGA analysis thrice. The average weight loss of the organic component was 43.06 ± 0.26%. Then, the exact stoichiometry of the intercalated system sample was TiS_2_(HA)_0.833_, not far from the TiS_2_(HA) formula used as a model for our DFT simulations (vide infra).

**Figure 4:**
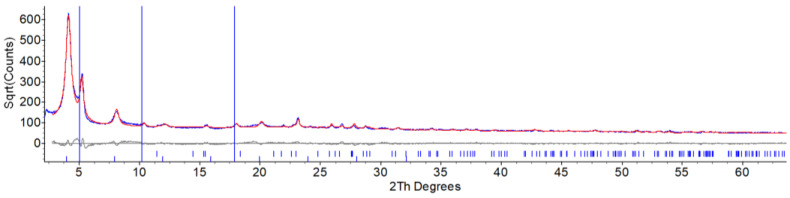
XRD pattern of 12-month-aged TiS_2_/HA, showing the partial degradation of the intercalated material with the formation of crystalline sulfur. Data modeling of the polyphasic TiS_2_/HA sample was performed by a hybrid approach comprising Rietveld refinement of rhombic sulfur and 1D structureless Le Bail refinement of the TiS_2_/HA species. Blue trace: observed data; red trace, simulated pattern. Difference plot (in grey) and peak markers, for sulfur and 00l reflections belonging to the TiS_2_/HA crystal phase (blue ticks), are drawn at the bottom. The three spurious peaks highlighted by the blue vertical lines are attributed to different HA packing (or content) and/or to unknown contaminants.

After 12 months of aging in the dark at environmental conditions, the XRPD pattern of powders of TiS_2_/HA evidenced the limited stability of this intercalated material. Moreover, the formation of new crystalline phases was clearly observed. Indeed, jointly with residual TiS_2_/HA, elemental sulfur was formed in its low-temperature/low-pressure orthorhombic polymorph (*Fddd* space group [33]). The complete Le Bail/single peak and Rietveld refinement plot for such sample, which contains TiS_2_/HA, an unknown contaminant and crystalline sulfur, is shown in Figure 4.

A puzzling and open question, however, remains: what is the fate of titanium atoms? As anticipated, sulfur exsolution is accompanied by the formation of an unknown and partially crystalline contaminant (see blue vertical lines in Figure 4). The few uninterpreted diffraction peaks do not match any titania polymorph, nor could they be related to any other reasonable reaction product, such as those presented in the computational study discussed below, with the obvious substitution of NH_3−n_ residues with CH_3_(CH_2_)_5_NH_2−n_ ones (*n* = 0, 1, 2). Additionally, the large incoherent scattering raising the overall background level suggests that noncrystalline components are present (amorphous titania and its congeners [34], to mention a few).

**Figure 5:**
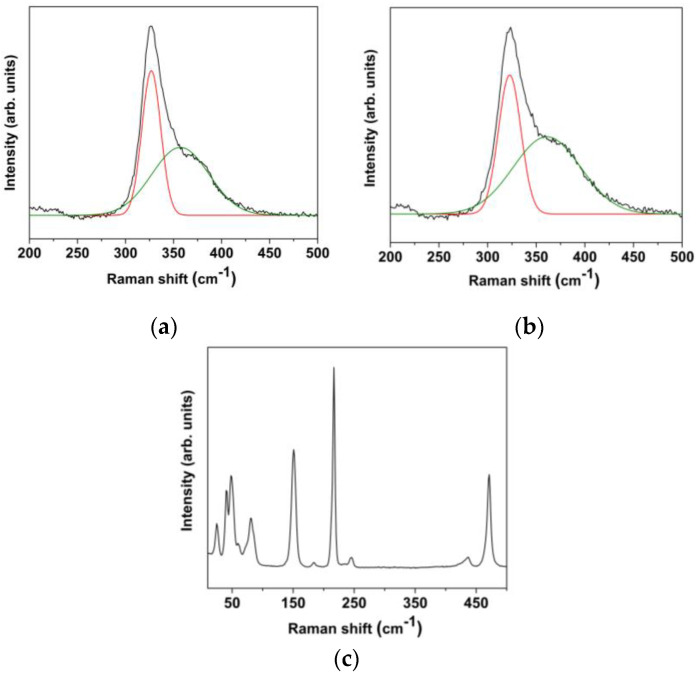
Micro-Raman spectra of (**a**) pristine TiS_2_, (**b**) TiS_2_/HA, and (**c**) aged TiS_2_/HA. In panels (**a**,**b**), the deconvolution of the Raman peaks with two components is shown.

Further evidence of sulfur demixing is given by Raman spectroscopy. Figure 5 shows the Raman spectra of three samples: pristine TiS_2_ and TiS_2_/HA (in the 200–500 cm^−1^ range) and aged TiS_2_/HA (20–500 cm^−1^). As expected, the Raman spectrum of the as-prepared TiS_2_/HA is very similar to that of pristine TiS_2_, while the aged TiS_2_/HA hybrid exhibits many more signals due to sulfur exsolution.

The phonon modes at the Γ-point of the reciprocal space can be probed by Raman and IR spectroscopies. The primitive cell of TiS_2_ consists of two chalcogen atoms and one metal atom with trigonal prismatic coordination, with the optical normal modes of vibration *A*_1*g*_, *E_g_* (Raman active), *A*_2*u*_, and *E_u_* (IR active). The experimental Raman spectrum was then interpreted through simulation of the active modes (Table 1).

The calculated Raman shifts are in very good agreement with the experimental ones. In particular, the Raman-active mode out of the plane ($A_{1g}$) is measured at 326.9 cm^−1^ for the pristine system, and 322.7 cm^−1^ for the intercalated one, confirming that the simulation captures well the electronic structure of the layered compound.

**Figure 6:**
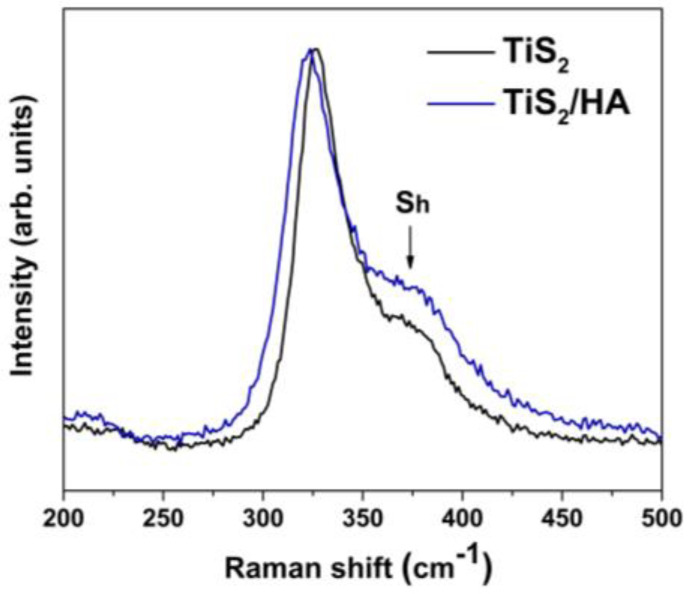
Comparison between the Micro-Raman spectra of pristine TiS_2_ (black) and of the intercalated TiS_2_/HA hybrid (blue).

A high-energy shoulder peak located at ~360 cm^−1^, labeled Sh in the literature, is evident in the Raman spectra as an additional component (shown in Figure 6, TiS_2_ and TiS_2_/HA). Although this peak position matches the calculated frequency of the $A_{2u}$ mode, it cannot be ascribed to this vibrational mode, the symmetry of which (*A*_2*u*_) makes it only IR active. The physical origin of this shoulder has been subject to several interpretations in the literature, but no general consensus has been reached to date [35,36,37]. Significantly, the presence of intercalated HA between the Ti-S layers does not affect the vibration modes in the measured range (see Figure 6).

**Figure 7:**
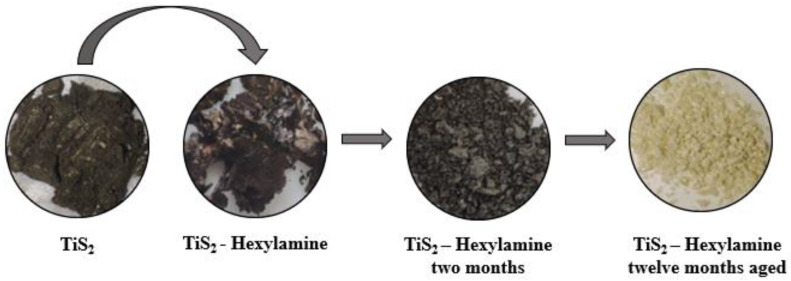
Macroscopic morphological and color changes of TiS_2_/HA.

**Figure 8:**
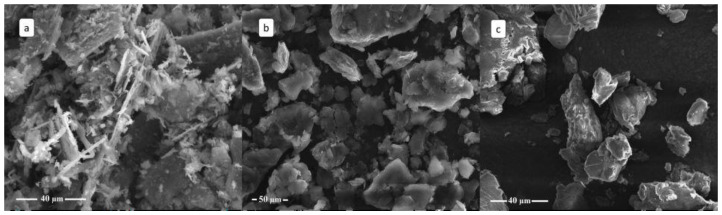
SEM images of (**a**) pristine TiS_2_, (**b**) fresh TiS_2_/HA, and (**c**) aged TiS_2_/HA.

An interesting aspect of the change brought about by the intercalation process is the morphological evolution of the sample with time, evident at both the macroscopic and the microscopic scales. As Figure 7 shows, pristine TiS_2_ is a fine and dry black powder, while TiS_2_/HA is a brown sticky powder, probably because of residual (not intercalated) hexylamine. Due to progressive material degradation, after 2 months, TiS_2_/HA turns almost grey, and 1 year later, a yellow powder is found instead. The morphology of TiS_2_, freshly prepared and aged (in air) TiS_2_/HA hybrids, was also investigated using SEM imaging (Figure 8).

The SEM images of the intercalated compounds (both fresh and aged) show a substantial modification of the TiS_2_ morphology. In pristine TiS_2_, the lamellar shape of the crystals is evident, but it fully disappears in the freshly prepared TiS_2_/HA hybrid, demonstrating that the mechanical treatment induces a severe morphological change, where a simple topotactic HA insertion does not maintain crystal size and shapes, it being accompanied by a complete lamellar disruption. Estimation of the average crystal domain lengths (along c) from the peak broadening of XRPD data confirms that from micrometer-sized TiS_2_ crystals, coherent domains of average size as low as 40 nm are formed in TiS_2_/HA. On the other hand, there is no substantial microscopic modification upon aging, despite the occurrence of sulfur exsolution (with coherent isotropic domains of about 50 nm) and partial sample degradation. The hybrid structure, obtained by intercalation of the HA within the TiS_2_ lattice, remains stable even after partial sulfur elimination. The hypothesis is confirmed by the XRPD pattern reported above in Figure 4, as the persistence of the 001 peak of the intercalated compound is the dominating signal also upon material aging.

In order to pinpoint the driving force behind the decomposition of TiS_2_/HA, and a possible mechanism for sulfur exsolution, we calculated the enthalpy variations of several possible reactions (ΔH_r_, as E(products)—E(reactants)) with the DFT + U scheme described above. As is standard practice in this kind of simulations, the difference in total energy of products and reactants is taken as an approximation of the enthalpy of reaction.

**Figure 9:**
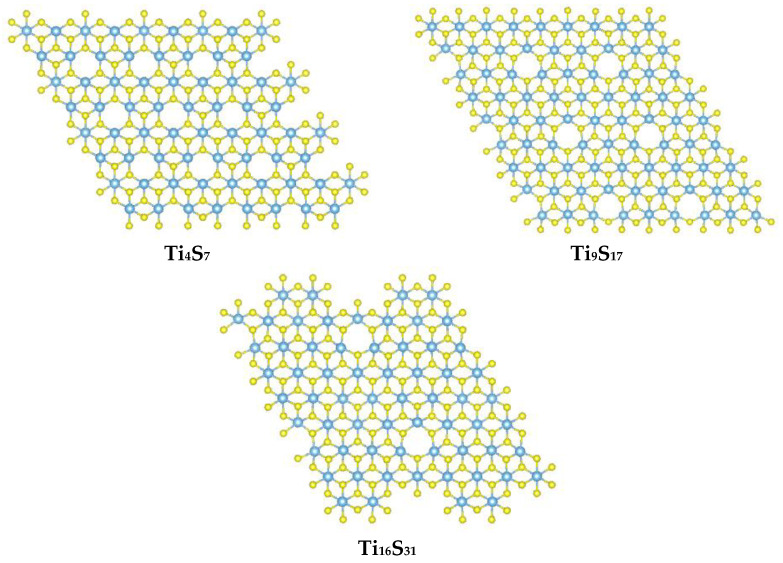
Top view of the different TiS_2_ supercells used in the DFT modeling, all with periodic sulfur vacant sites.

Initially, different sulfur-deficient TiS_2_ superstructures were modeled. Three progressively larger supercells were constructed, with a corresponding dilution of sulfur vacancies. These ideal structures are synoptically depicted in Figure 9. As shown in Table 2, the enthalpic cost for the formation of a sulfur vacancy decreases as the cell size increases, but in all cases, the formation of such vacant sites is never energetically favored per se. Using a simple linear extrapolation of the ΔH_r_ with 1/*n* plot (for the Ti_n_S_m_ formulation), the energy required to form an infinitely diluted (neutral) S-vacancy in TiS_2_ at the bulk limit can be estimated to be ca. 2.2 eV.

Once the stability of pristine TiS_2_ was quantitatively confirmed, to understand and interpret the experimentally observed spontaneous intercalation of HA, we started considering two consecutive steps: (i) the formation of the TiS_2_/HA hybrid and (ii) sulfur exsolution therefrom.

**Figure 10:**
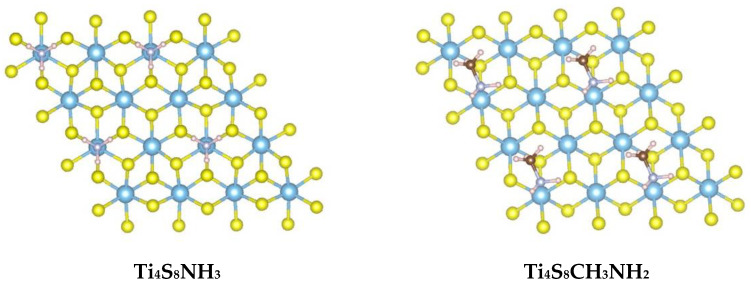
[001] view of NH_3_ and CH_3_NH_2_ molecules adsorbed onto a TiS_2_ slab containing 16 metal atoms. Nitrogen atoms interact vertically with the Ti atoms of the TiS_2_ surface, with N…Ti distances of around 2.25 Å.

To reduce computational costs, we investigated the intercalation, within TiS_2_, of simple amine molecules (using ammonia and methylamine instead of HA) (Table 3) with an idealized coverage of one nitrogen atom per four titanium ions (see Figure 10). Our calculations were then based on 2 × 2 TiS_2_ supercells with a single-side N-substitution. The intercalation reaction was mediated by moisture as amines or ammonia were used as aqueous solutions.

**Figure 11:**
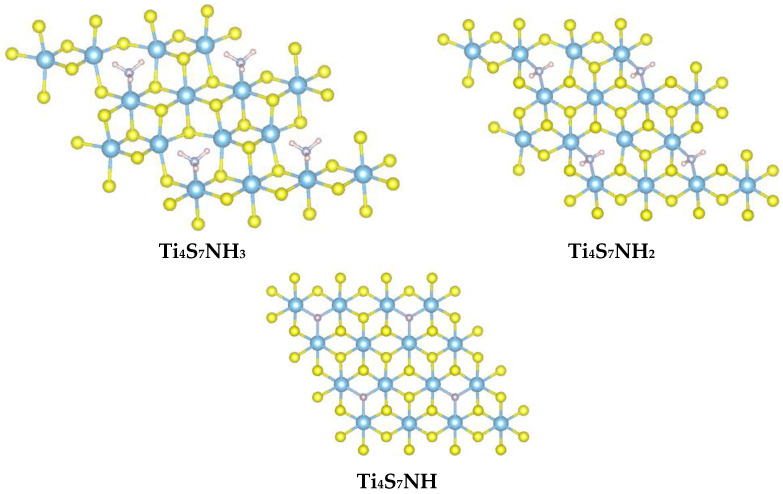
Top view of the nitrogen-containing Ti-S slabs formed by the loss of S atoms, substituted by ammonia molecules. In the last panel, all H atoms are eclipsed by N atoms, as N-H bonds lie perpendicular to the plane of drawing.

That the intercalation reaction, observed experimentally for hexylamine, is energetically favored is here confirmed by the simulated reaction energies, which are negative for both ammonia and methylamine cases. Once these adducts are formed, one sulfur atom is lost and nitrogen from the amine replaces it, with the formation of a Ti-N interaction. The fact that the NH_3_ molecule interacts favorably with Ti, replacing S in its position, suggests that the Ti-N bond formation may drive S exsolution. For this reason, a few different possible reactions were tested, all involving the formation of defective TiS_2_ slabs where amine groups carrying one to three H atoms actively interact with open Ti sites in close proximity to a sulfur vacancy. Some of these complexes are shown in the case of ammonia (Figure 11), where the S atom is replaced by N. A similar situation is obtained in the case of methylamine.

The last row in Table 4 indicates that the substitution of S by N is probably mediated by the action of molecular oxygen. In other words, the intercalated TiS_2_/amine compounds seem to undergo sulfide oxidation to elemental sulfur, accompanied by a structural change in which Ti-N bonds are formed. In particular, the imido Ti_4_S_7_NH structure, where N is bound to three Ti sites, appears to be the most stable. Such μ_3_-NH coordination geometry on three Ti ions should not surprise, as it was found in a number of polynuclear organometallic complexes and, more paradigmatically, also in the highly symmetric [Ti(NH)(Cp*)]_4_ cubane (Cp* = tetramethyltrimethylsilylcyclopentadiene) [38].

However, it should be noted that all four reactions reported in Table 4 are in principle consistent with the available experimental evidence (exsolution of crystalline sulfur). The impossibility of verifying experimentally which is the true path to sulfur elimination (which likely occurs very locally without the formation of a clearly distinguishable crystalline phase) makes uncertain the nature, stoichiometry, and structure of the newly formed Ti/S/N product.

In order to quantitatively assess the effective charge transfer between N and Ti, the electron densities (in the form of Bader charges [39,40]) of pristine TiS_2_ and different Ti-N complex supercells were compared (Table 5). By comparing bound structures with varying amounts of H atoms, it is seen that the Ti-N charge transfer is more and more effective as the amine group loses hydrogen atoms and forms stronger bonds with titanium: the Ti electron density decreases and the N/H electron density increases.

**Figure 12:**
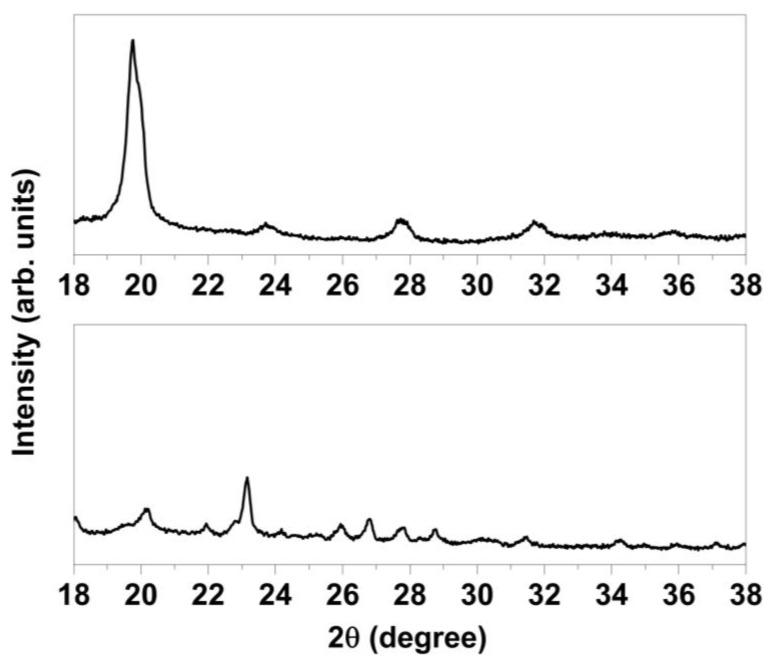
XRPD patterns of TiS_2_/HA (N_2_) (**top**) and TiS_2_/HA (air) (**bottom**), showing that sulfur exsolution only occurs by exposure to molecular oxygen.

To experimentally test the hypothesis that the structural change (and material decomposition) of TiS_2_/HA was a consequence of exposure to molecular oxygen, a newly prepared batch of TiS_2_/HA was kept under inert atmosphere, while another batch was kept in air for comparison (both in the dark and at room temperature, for 1 week). Figure 12 compares the XRPD patterns of these two samples. The absence of crystalline sulfur in the case of TiS_2_/HA kept in inert atmosphere is the experimental confirmation of the proposed reaction pathway, where O_2_ actively participates in sulfur elimination.

## 4. Conclusions

We report experimental evidence of limited stability in ambient conditions of hexylamine-intercalated titanium disulfide, a material that has recently attracted interest for prospected thermoelectric applications. X-ray powder diffraction and Raman spectroscopy clearly show the formation of crystalline sulfur at the expense of the layered TiS_2_/hexylamine compound. Different degradation mechanisms were tested by ab initio periodic DFT calculations on a TiS_2_/NH_3_ model system, suggesting that TiS_2_ reacts with molecular oxygen, resulting in Ti-N adducts, where N resides in lieu of S vacancies, with the subsequent formation of elemental sulfur and water. A comparison of the aging of TiS_2_/HA in the presence or the absence of oxygen further corroborates the proposed mechanism, since prolonged storage in inert atmosphere does not result in the degradation of the intercalated material. Since our findings point to a limited stability in air of such hybrid systems, appropriate actions taken to minimize exposure to oxygen in practical applications should be devised for TMDC to be used in environmental conditions. While this appears to be a severe limitation for their wide usage, similar issues have been recently solved for oxygen- and moisture-labile hybrid materials (lead halide perovskites, above all [41]), which are deposited within thin layers of nonpermeable, and optically transparent, plastic films.

## Figures and Tables

**Table 1:** Vibrational modes of TiS_2_ calculated by DFT + U simulations.

Mode	Raman Shift (cm^−1^)
*E_u_*	162.45
*E_g_*	233.10
*A*_1*g*_	321.23
*A*_2*u*_	361.10

**Table 2:** Formation of sulfur defects in TiS_2_ at different concentrations (down to 0.03 at%). Extrapolation to an infinitely diluted vacant site leads to 2.2 eV, which is the energy required to eliminate one single (neutral) S atom from bulk TiS_2_.

Reaction	ΔH_r_ (eV)
Ti_4_S_8_ → Ti_4_S_7_ + S	3.14
Ti_9_S_18_ → Ti_9_S_17_ + S	2.71
Ti_16_S_32_ → Ti_16_S_31_ + S	2.43

**Table 3:** Reaction energy of TiS_2_ intercalation compounds with either aqueous ammonia or aqueous methylamine.

Reaction	ΔH_r_ (eV)
Ti_4_S_8_ + NH_4_^+^ + OH^−^ → Ti_4_S_8_NH_3_ + H_2_O	−2.93
Ti_4_S_8_ + CH_3_NH_3_^+^ + OH^−^ → Ti_4_S_8_CH_3_NH_2_ + H_2_O	−1.75

**Table 4:** Reaction energy of the formation of Ti-N bonds arising from the exsolution of S and its substitution with the (deprotonated) ammonia molecules.

Reaction	ΔH_r_ (eV)
Ti_4_S_8_NH_3_ → Ti_4_S_7_NH_3_ + S	1.51
Ti_4_S_8_NH_3_ → Ti_4_S_7_NH_2_ + S + ½ H_2_	1.47
Ti_4_S_8_NH_3_ → Ti_4_S_7_NH + S + H_2_	1.76
Ti_4_S_8_NH_3_ + ½ O_2_ → Ti_4_S_7_NH + S + H_2_O	−0.83

**Table 5:** Bader charges of TiS_2_ supercell and the Ti-N supercells formed via sulfur exsolution and substitution with nitrogen. The number of electrons explicitly included in the calculation is indicated for each element.

	Ti_4_S_8_	Ti_4_S_7_NH_3_	Ti_4_S_7_NH_2_	Ti_4_S_7_NH
**Ti (10)**	10.23	10.2721	10.1974	10.1304
**S (8)**	6.89	6.9950	6.9530	6.9218
**N (7)**	-	6.2964	6.3866	6.4353
**H (1)**	-	0.5500	0.5760	0.5900
**Total Ti/S**	96.00	90.05	89.46	88.97
**Total ammonia**	-	7.95	7.54	7.03
**Total**	96.00	98.00	97.00	96.00

## Data Availability

The data presented in this study are available on request from the corresponding author.

## Supplementary Materials

The following supporting information can be downloaded at: https://www.mdpi.com/article/10.3390/ma15238297/s1, Figure S1: Rietveld refinement of the pristine TiS_2_ material; Figure S2: Structureless 1D Le Bail refinement of TiS_2_/HA hybrid with 00l peaks only; Figure S3: Structureless 1D Le Bail refinement of TiS2/HA (N_2_).
