# Supplementary material for: Long-Term Stability of TiS2–Alkylamine Hybrid Materials
Source: Materials (Basel). 2022 Nov 22;15(23):8297. doi: 10.3390/ma15238297 (PMC9736076; doi:10.3390/ma15238297)
Supplement: Supplementary file 1 [file materials-15-08297-s001.zip › materials-2019064-supplementary.pdf]

Long-term stability of TiS_2-alkylamine hybrid materials

Federica Ursi, Simone Virga, Gonzalo Garcìa-Espejo, Norberto Masciocchi, Antonino Martorana, Francesco Giannici

SUPPORTING INFORMATION

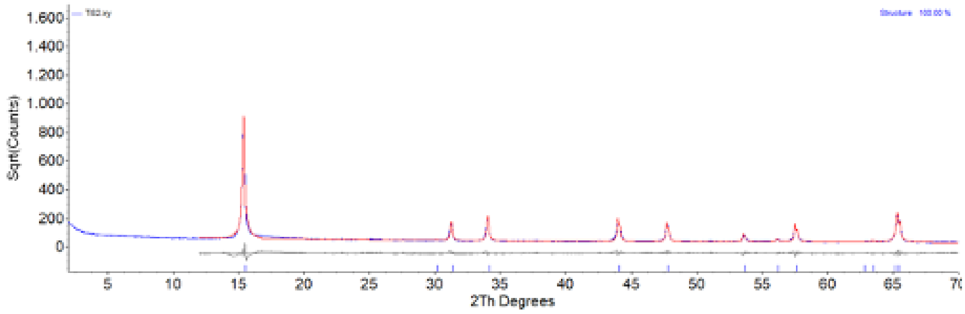

Figure S1 - Rietveld refinement of the pristine TiS_2 material. Y-axis is in square root scale. All experimental peaks belong to the titanium disulfide structure. XRD modeling required the insertion of a strong textural component, with the preferential orientation parameter r_{001} = 0.42 (in March-Dollase desctiption,).

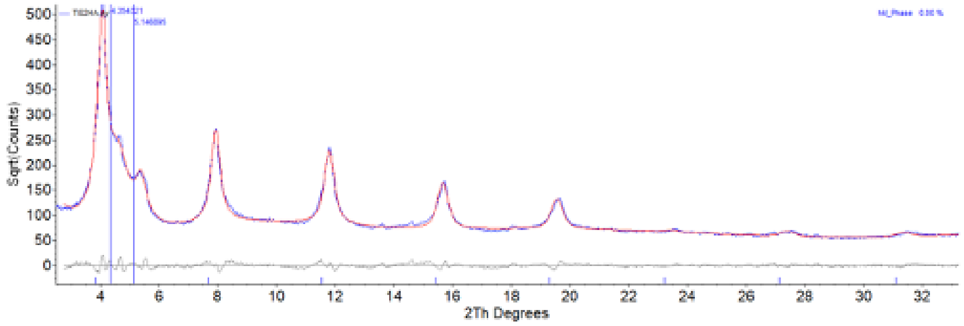

Figure S2 - Structureless 1D Le Bail refinement of TiS_2/HA hybrid *with 00l peaks only*. Y-axis is in square root scale. These experimental peaks are typical of an inflated titanium disulfide structure, with a significantly large interlayer spacing (23.0 Å, vs. 5.7 Å of the original TiS_2 powders). Two additional peaks (vertical lines at 4.35-5.14°) are attributed to minor contaminants characterized by different packings of HA molecules in the interlayer space, or, more likely, to a slightly lower amount of intercalated HA moieties.

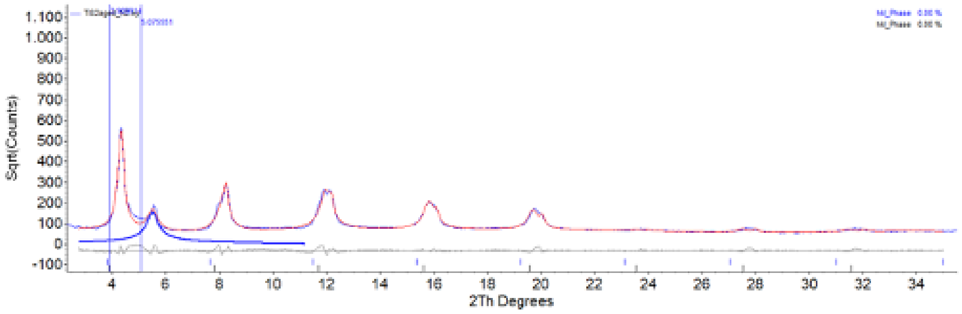

Figure S3 - Structureless 1D Le Bail refinement of TiS2/HA (N_2). Y-axis is in square root scale. No significant differences with respect to TiS_2/HA. are evident. As explained in the main text, the spurious peak highlighted by the blue trace is attributed to different HA packing (or content).
